# Introducing the Blastocyst Fragmentation Indicator (BFI): A Novel Time-Lapse Metric for Enhanced Aneuploidy Risk Stratification in Non-Invasive Embryo Assessment

**DOI:** 10.5935/1518-0557.20250171

**Published:** 2026

**Authors:** Hamilton de Martin, Eduardo Gomes Sá, Andrea Mesquita Lima, Ellayne Cavalcanti Queiroz, Gleicyane Sousa dos Santos Alam, Tulius Augustus Ferreira de Freitas, Gabriel Acácio de Moura, Eduardo de Paula Miranda, Sebastião Evangelista Torquato

**Affiliations:** 1 Bios Human Reproduction Center, Clínica Evangelista Torquato, Fortaleza, Ceará, Brazil

**Keywords:** Time-lapse monitoring, embryo selection, blastocyst fragmentation, embryo grading, chromosomal abnormalities

## Abstract

**Objective:**

To propose an improved blastocyst grading system by integrating dynamic time-lapse parameters with conventional morphological criteria, aiming to enhance embryo selection during assisted reproduction.

**Methods:**

This retrospective cohort study evaluated 1,182 embryos derived from 433 cycles of intracytoplasmic sperm injection. Time-lapse parameters included morula compaction status, blastocyst collapse frequency, and fragmentation of the inner cell mass and trophectoderm. These findings were analyzed in conjunction with standard morphological grading and embryo scoring systems based on implantation data. Embryo outcomes were stratified by chromosomal status determined through preimplantation genetic testing for aneuploidy.

**Results:**

Fully compacted morulae were associated with higher morphological grades; however, the proportion of chromosomally normal embryos did not differ significantly when compared to those derived from partially compacted morulae, suggesting that static morphology alone may not accurately reflect developmental potential. Although most blastocyst collapse events were minor, they correlated with increased fragmentation and reduced embryo quality. Fragmentation of the inner cell mass and/or the trophectoderm was associated with significantly lower rates of chromosomal normalcy. Logistic regression analysis confirmed fragmentation as the strongest predictor of aneuploidy, outperforming both conventional grading systems.

**Conclusions:**

This integrated evaluation model, combining static morphology with dynamic developmental events, offers a more comprehensive and accurate approach to assessing embryo viability. By identifying embryos with higher risk of chromosomal abnormalities, this strategy may enhance embryo selection, improve reproductive outcomes, and contribute to more individualized care in assisted reproduction.

## INTRODUCTION

The morphological evaluation of embryos, particularly at the blastocyst stage, is pivotal in embryo selection for transfer during in vitro fertilization (IVF) cycles. The Gardner and Schoolcraft grading system (1999) remains widely used for this purpose (Gardner & Schoolcraft, 1999), offering a morphological scoring system based on blastocyst expansion and the quality of both the inner cell mass (ICM) and the trophectoderm (TE). While effective, this approach has limitations due to its static and subjective nature, as it assesses embryos at specific time points without accounting for dynamic events that can influence implantation potential (ALPHA Scientists in Reproductive Medicine & ESHRE Special Interest Group of Embryology, 2011).

In recent years, time-lapse imaging technology has introduced a new dimension to embryo evaluation, enabling continuous observation of morphokinetic parameters in real time. Studies have demonstrated that incorporating dynamic data, such as the timing and synchrony of cell divisions, improves implantation success predictions compared to static morphological evaluation alone (Meseguer *et al.*, 2011).

Time-lapse studies have shown that the timing and completeness of compaction are strong indicators of developmental potential (ESHRE Working group on Time-lapse technology *et al.*, 2020). Fully compacted morulae are generally more likely to progress to high-quality blastocysts with robust chromosomal profiles, while partial or delayed compaction patterns are associated with reduced rates of blastocyst formation (Hur *et al.*, 2023). Nevertheless, recent findings suggest that partially compacted morulae that do reach the blastocyst stage can exhibit euploidy rates comparable to those of fully compacted morulae. Counterintuitively, this indicates that chromosomal integrity can be maintained even in the presence of incomplete compaction (De Martin *et al.*, 2024). These findings highlight morula compaction as a meaningful developmental milestone with predictive value for embryo selection.

Another crucial parameter enabled by time-lapse monitoring is blastocyst collapse, a transient reduction in blastocoel volume followed by re-expansion. Controlled collapse and rapid recovery may be indicative of natural cellular reorganization, while frequent collapses or delayed recovery are often markers of compromised viability (Sciorio & Meseguer, 2021; Cimadomo *et al.*, 2022). These findings underline the value of monitoring collapse events and recovery patterns as indicators of developmental potential.

Finally, ICM and TE fragmentation are essential factors in embryo viability. The ICM, which forms fetal tissues, and the TE, which contributes to placental development, are critical to embryo competence. Excessive fragmentation within these regions may signal reduced developmental potential, with ICM fragmentation affecting fetal development and TE integrity influencing implantation success (Pribenszky *et al.*, 2017; Ozguldez *et al.*, 2020; Ai *et al.*, 2021).

This study prioritizes the evaluation of ploidy status over pregnancy outcomes, in line with the recommendations of Bamford *et al*. (2023). We propose an integrative blastocyst grading system that complements the conventional Gardner classification by incorporating dynamic and advanced morphological parameters. Specifically, this study introduces inner cell mass (ICM) and trophectoderm (TE) fragmentation as a novel parameter, alongside morula compaction quality and blastocyst collapse frequency. By combining classical morphology assessment with these critical developmental events, our approach seeks to refine embryo selection in IVF and optimize clinical outcomes.

## MATERIALS AND METHODS

### Study Design

This retrospective cohort study is based on anonymized data from embryos generated by in vitro fertilization (IVF) and cultured in a time-lapse system. All data were collected as part of frozen embryo transfer (FET) IVF cycles performed routinely at a single assisted reproduction center. Only data from cycles involving ovarian stimulation, oocyte retrieval, fertilization via intracytoplasmic sperm injection (ICSI), embryo culture in a time-lapse incubator, and blastocyst biopsy for preimplantation genetic testing for aneuploidy (PGT-A) were included in the study. Written informed consent was obtained from all patients, authorizing the anonymous use of clinical and laboratory data for research purposes.

### Data Collection

Data were obtained from 396 patients treated at a single assisted reproduction center. All clinical procedures followed the guidelines of the ASRM, ESHRE, SBRA, and CFM. The database includes 1182 embryos from 433 ICSI cycles performed between September 2022 and December 2024. Inclusion criteria were as follows: (i) embryos individually cultured and monitored from the zygote to blastocyst stage in a time-lapse incubator (EmbryoScope Plus, Vitrolife, Denmark); (ii) blastocysts analyzed by next-generation sequencing (NGS) following a single trophectoderm biopsy. The primary indication for IVF was advanced maternal age (≥ 37 years in 54.8% of cases), with unexplained infertility (11.3%), male factor (23.5%), and other factors (10.4%) accounting for the remainder.

### Time-Lapse Monitoring and Assessment

Embryos were cultured in a time-lapse incubator, allowing continuous monitoring without disturbance to the culture environment. Developmental timing parameters were automatically recorded (for descriptive purposes only) and included the time to pronuclear fading (tPNf), 2-cell division (t2), 4-cell division (t4), 5-cell division (t5), 8-cell division (t8), morula stage (tM), onset of cavitation (tSB), full blastocyst formation (tB) and expanded blastocyst formation (tEB).

During monitoring, spontaneous collapse events were recorded and classified as either minor collapses or major collapses, with total counts computed for each type. Additionally, the presence or absence of fragmentation in the inner cell mass (ICM) and trophectoderm (TE) was noted, with N (Normal) indicating no fragmentation and F (Fragmented) indicating the presence of fragmentation. Embryos were then categorized into three groups based on the integrity of these structures: NN: ICM and TE intact (no fragmentation); FN: Fragmented ICM with intact TE; and FF: Fragmentation in both ICM and TE.

**Figure 1:**
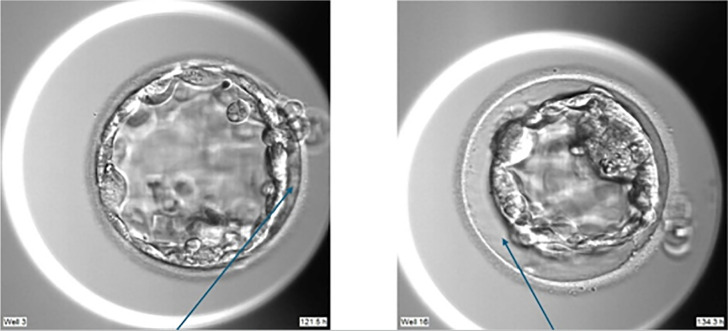
Spontaneous collapses: CL (Minor Collapse); MCL (Major Collapse).

Figure 1 illustrates examples of embryos exhibiting ‘minor’ and ‘major’ collapses. A ‘minor collapse’ is defined by a localized or incomplete retraction of the trophectoderm (TE) away from the zona pellucida. A ‘major collapse’, conversely, describes a complete separation or detachment of the trophectoderm (TE) from the entire inner surface of the zona pellucida.

**Figure 2:**
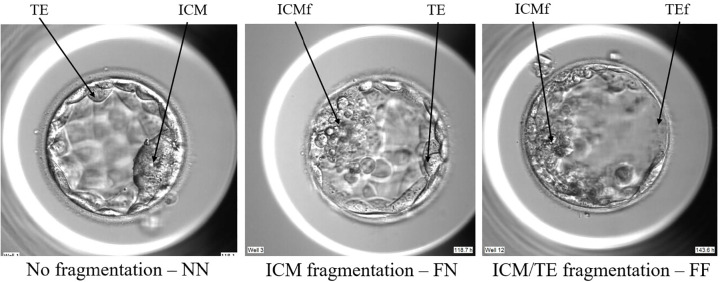
ICM/TE fragmentation.

Figure 2 illustrates embryos exhibiting different patterns of ICM/TE fragmentation. Embryos designated as FN are characterized by fragmentation that is either exclusive to, or primarily located in, the inner cell mass (ICMf). In contrast, embryos classified as FF display fragmentation involving both the inner cell mass (ICMf) and the trophectoderm (TEf).

**Figure 3:**
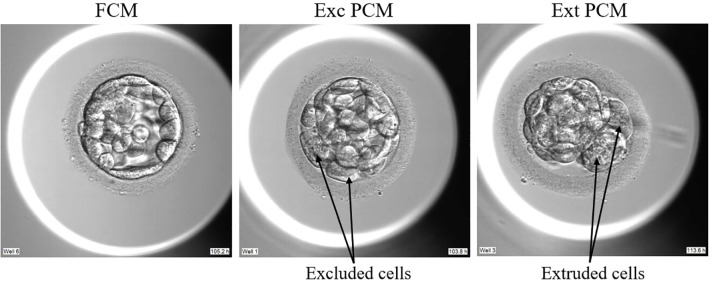
Morula compaction.

In addition, blastocyst morphology was classified using the Gardner system and categorized into groups A (comprising AA, AB, BA), B (BB, BC, AC), and C (CA, CB, CC). KIDScore categories (KIDScore D5 v3.1) were defined as A (KIDScore ≥7), B (KIDScore 4-7), and C (KIDScore <4). Morula compaction was assessed and categorized as fully compacted (FCM), partially compacted with excluded cells (Exc-PCM), or partially compacted with extruded cells (Ext-PCM). This classification, which is illustrated in Figure 3, follows the criteria described by Lagalla *et al.* (2017).

### Genetic Analysis and Morphological Classification

Blastocysts were assessed for biopsy eligibility on days 5, 6, or 7 of development. Internal diameters were measured using EmbryoViewer software (Vitrolife). Trophectoderm biopsy, restricted to fully expanded blastocysts (diameter >160 µm), involved laser-assisted herniation to facilitate retrieval of 5-7 cells. Retrieved blastomeres underwent preimplantation genetic testing for aneuploidy (PGT-A) via next-generation sequencing to determine ploidy status. Post-biopsy, embryos were vitrified pending PGT-A results.

### Statistical Analyses

Categorical variables were summarized as absolute numbers and percentages, and numerical variables as means, standard deviations (SD), and ranges. Trends in categorical variables were analyzed using the chi-square test. A Generalized Linear Model (GLM) with Poisson distribution assessed the association between ICM/TE fragmentation and blastocyst collapse frequency (minor/major). Logistic regression, controlling for maternal age, examined the association between blastocyst classification criteria (Gardner, KIDScore, and ICM/TE fragmentation) and ploidy status, and odds ratios (ORs) with 95% confidence intervals (CIs) were calculated. Statistical analyses were performed with JAMOVI software (version 2.6.13), with *p*<0.05 considered significant.

## RESULTS

**Figure 4:**
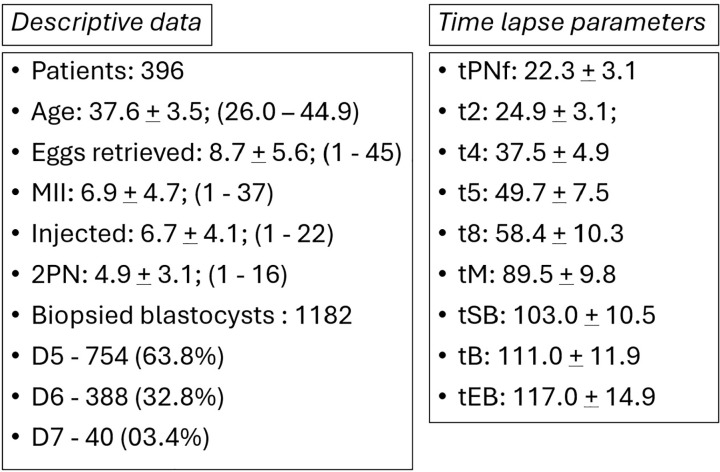
Descriptive data and time-lapse parameters.

This study evaluated 1182 embryos from 433 ICSI cycles (396 patients) reaching the blastocyst stage, subsequently biopsied for PGT-A. Maternal age ranged from 26.0 to 44.9 years (mean 37.6 ± 3.5 years). Analyses were performed on morula compaction patterns, morphology (Gardner’s criteria), morphokinetics (KIDScore), spontaneous collapse events, and ICM/TE fragmentation, stratified by maternal age: <37 years (45.6%) and >37 years (54.4%). Descriptive data are summarized in Figure 4. The overall euploidy rate was 44.5%, with 54.7% and 35.9% observed in patients aged <37 and >37 years, respectively.

### Spontaneous Collapses

**Figure 5:**
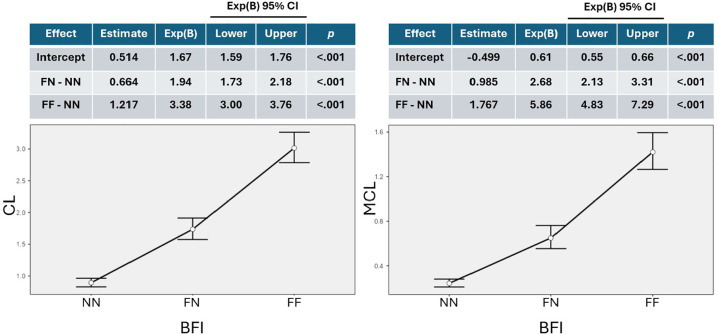
Frequency of Minor and Major Blastocyst Collapses According to Fragmentation Pattern. Generalized linear models (Poisson distribution with Bca bootstrap, 1,000 replications) were used to estimate the frequency of minor (CL, left panel) and major (MCL, right panel) collapse events in blastocysts, based on the distribution of inner cell mass (ICM) and trophectoderm (TE) fragmentation. Embryos were classified into three categories: NN (no fragmentation), FN (fragmentation restricted to the ICM), and FF (fragmentation in both ICM and TE).

Minor collapses occurred in 69.0% of blastocysts, while major collapses were identified in 34.3% of embryos. Logistic regression analysis demonstrated a significant association between collapse events and euploidy (*p*<0.001); however, predictive power was limited, with AUC values of 0.597 for minor collapses and 0.588 for major collapses. Non-fragmented embryos (NN) exhibited the lowest frequencies of minor (0.893) and major collapses (0.243), which increased in partially fragmented (FN, minor = 1.735, major = 0.650) and fully fragmented embryos (FF, minor = 3.015, major = 1.421). GLM analysis (Figure 5) confirmed that ICM/TE fragmentation significantly increased the prevalence of blastocyst collapse. Compared to NN, FN embryos had 1.94- and 2.68-fold higher prevalence of minor (CL) and major (MCL) collapses, while FF embryos showed 3.38- and 5.86-fold increases (Figure 5).

### Morula Compaction and Euploidy

**Figure 6:**
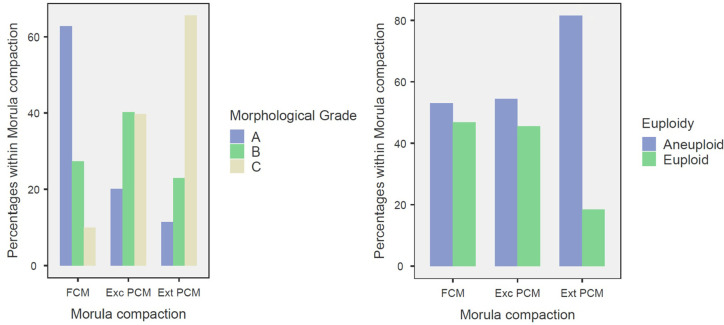
Morphological Scores and Euploidy Rates According to Morula Compaction Patterns. Distribution of morphological grades (A, B, C) (left panel) and euploidy status (euploid vs. aneuploid) (right panel) across three morula compaction categories: fully compacted morulae (FCM), partially compacted morulae with excluded cells (Exc PCM), and partially compacted morulae with extruded cells (Ext PCM).

Fully compacted morulae (FCM) accounted for 73.7% of the embryos analyzed, with 62.7% reaching Gardner grade A. In contrast, partially compacted morulae with excluded cells (Exc PCM) comprised 19.0% of the cohort, and only 20.1% were classified as grade A. Despite these marked differences in morphological quality, the euploidy rates were comparable between FCM (46.8%) and Exc PCM (45.5%), as shown in Figure 6. Notably, the majority of blastocysts exhibiting fragmentation restricted to the inner cell mass (FN) originated from FCM (70.9%), whereas only 21.8% derived from Exc PCM. A similar pattern was observed among blastocysts displaying concurrent fragmentation of both the inner cell mass and trophectoderm (FF), with 50.5% arising from FCM and 24.8% from Exc PCM.

### Comparison of Selection Criteria

**Figure 7:**
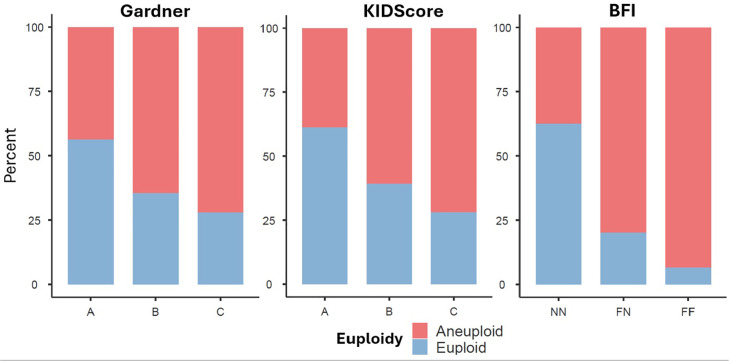
Euploidy Rates According to Gardner Grading, KIDScore, and the Blastocyst Fragmentation Indicator (BFI). Stacked bar plots showing the proportion of euploid (blue) and aneuploid (red) embryos across morphological grades (A, B, C) using the Gardner system (left panel), the KIDScore classification (middle panel), and the BFI categories (right panel): NN (no fragmentation), FN (fragmentation in the inner cell mass), and FF (fragmentation in both the inner cell mass and trophectoderm). The figure illustrates the distribution of chromosomal status within each scoring system.

Euploidy rates increased alongside improvements in both Gardner grading (A: 56.2%, B: 35.3%, C: 27.9%) and KIDScore (grade A: 61.1%, B: 39.2%, C: 28.0%). However, KIDScore classified fewer embryos as grade A compared to the Gardner system (37.0% *vs*. 50.8%). In contrast, ICM/TE fragmentation exhibited the most predictive power for euploidy: non-fragmented (NN) embryos showed a 62.5% euploidy rate, whereas this was 20.1% for partially fragmented (FN), and only 6.4% for fully fragmented (FF) embryos. The contrasting patterns, including the much sharper decline in euploidy associated with ICM/TE fragmentation, are clearly presented in Figure 7.

**Figure 8:**
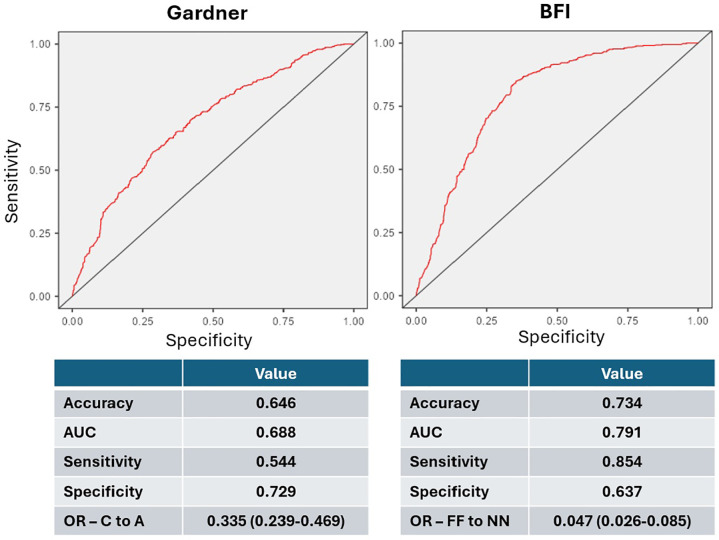
ROC Curves and Performance Metrics Comparing Gardner Grading and ICM/TE Fragmentation (BFI). Receiver operating characteristic (ROC) curves (top) and corresponding diagnostic metrics (bottom) comparing the predictive performance of the Gardner grading system (left panel) and the Blastocyst Fragmentation Indicator (BFI) based on inner cell mass (ICM) and trophectoderm (TE) fragmentation (right panel). Performance measures include accuracy, area under the curve (AUC), sensitivity, specificity, and odds ratios (OR) for the lowest scoring categories (Gardner grade C vs. A; FF vs. NN in BFI).

Logistic regression, adjusted for maternal age, confirmed significant reductions in euploidy likelihood for lower Gardner grades (grade B: OR 0.467, 95% CI 0.353-0.619; grade C: OR 0.335, 95% CI 0.239-0.469) and KIDScore grades (B: OR 0.443, 95% CI 0.336-0.584; C: OR 0.278, 95% CI 0.201-0.386). However, ICM/TE fragmentation showed the most profound effect. Compared to non-fragmented embryos, FN and FF embryos showed an 84% (OR 0.157, 95% CI 0.110-0.225) and 95% (OR 0.047, 95% CI 0.026-0.085) reduction in euploidy likelihood, respectively. AUC values for predicting euploidy were 0.697 for KIDScore, 0.688 for Gardner grading, and 0.791 for ICM/TE fragmentation (Figure 8). Odds ratios for aneuploidy in the lowest categories were 3.593 (KIDScore), 2.986 (Gardner grading), and 21.036 (ICM/TE fragmentation).

### Association with Specific Types of Aneuploidy

**Figure 9:**
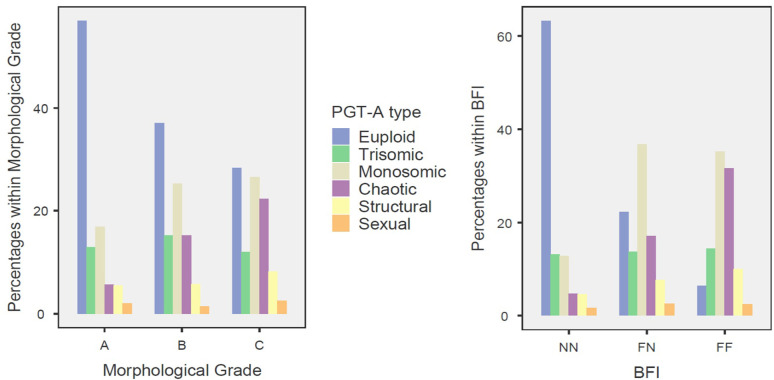
Distribution of PGT-A Aneuploidy Types According to Morphological Grade and BFI Classification. Stacked bar charts showing the relative distribution of preimplantation genetic testing for aneuploidy (PGT-A) results by aneuploidy type across morphological grades (left panel: A, B, C) and Blastocyst Fragmentation Indicator (BFI) categories (right panel: NN = no fragmentation, FN = fragmentation in inner cell mass, FF = fragmentation in both inner cell mass and trophectoderm).

Chi-squared analysis revealed a significant association between ICM/TE fragmentation and the type of aneuploidy (χ^2^ = 333.252, df = 10, *p*<0.001), with a graded increase in complex aneuploidies observed with greater fragmentation. Chaotic abnormalities were significantly more frequent in the FF group (31.7%) than the NN group (4.7%). Gardner morphological grading also showed a significant association with aneuploidy types (χ^2^ = 97.688, df = 10, *p*<0.001), but less pronounced than with fragmentation. Monosomies were more prevalent than trisomies in the FN (36.8%; 13,7%) and FF (35.1%; 14.4%) groups, whilst more balanced in NN embryos (12.7%; 13.1%). A striking discrepancy in aneuploidy patterns was found when comparing embryos classified by Gardner criteria (C) with those classified by ICM/TE fragmentation (FF), in contrast to the similarity between patterns observed for A and NN embryos (Figure 9).

**Figure 10:**
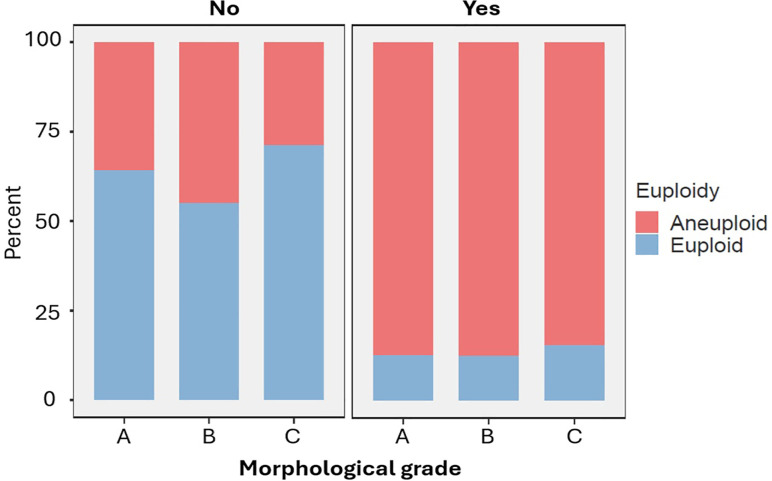
Euploidy Rates Across Morphological Grades According to the Presence or Absence of ICM/TE Fragmentation. Stacked bar plots representing the proportion of euploid (blue) and aneuploid (red) embryos across morphological grades A, B, and C, stratified by the absence (No, left panel) or presence (Yes, right panel) of fragmentation in the inner cell mass (ICM) and/or trophectoderm (TE). Each panel shows the chromosomal status distribution within each morphological category based on fragmentation status.

Analysis of euploidy rates within each morphological category (A, B, C) was conducted for embryos ‘without fragmentation’ (NN) versus ‘with fragmentation’ (combining FN and FF). Fragmentation had a significant negative impact on euploidy rates (13.8%) compared with those without fragmentation (62.5%; *p*<0.001). Moreover, no significant differences in euploidy rate were observed for grade A, B, and C embryos within the fragmented group (12.8%, 12.4%, and 15.5%, respectively; *p*=0.682). A non-significant trend for euploidy was observed within the non-fragmented group. This confirms that fragmentation, more than morphology alone, serves as a strong negative indicator for euploidy (Figure 10).

### Novel Classification System

A novel classification system was developed by integrating morula compaction status (FCM = 1, Exc-PCM = 2, Ext-PCM = 3) with ICM/TE integrity (N for normal, F for fragmented). The proposed nomenclature (e.g., 1-NN, 2-FN, 3-FF) enables a dynamic and predictive assessment of embryo quality. No blastocysts with an intact ICM and fragmented TE were observed. Minor and major collapses, whilst recorded by embryologists as supplementary information, are not included in the final classification. This system offers a robust tool for embryo selection, given the independence and predictive strength of ICM/TE fragmentation.

## DISCUSSION

This study introduces a simplified blastocyst ranking system based on time-lapse imaging parameters. The system integrates morula compaction, spontaneous blastocyst collapses, and, crucially, fragmentation of the inner cell mass (ICM) and trophectoderm (TE), providing a dynamic and detailed perspective of embryonic development to enhance embryo selection.

The single embryo transfer (SET) policy, widely adopted in assisted reproductive technology (ART) to minimize multiple pregnancy risks (Martin *et al.*, 2017; Practice Committee of the Society for Reproductive Endocrinology and Infertility, 2022), relies on selecting the most viable embryo. While the Gardner scoring system remains prevalent (Gardner & Schoolcraft, 1999), its correlation with ploidy is limited. Morphologically superior blastocysts are more likely to be euploid, but this is not definitive; up to half of morphologically excellent blastocysts may be aneuploid (Alfarawati *et al.*, 2011; Li *et al.*, 2022). This study observed euploidy rates of 56.2% for “A” graded embryos and 27.9% for “C” graded embryos, highlighting the risk of relying solely on morphology. Time-lapse imaging offers a dynamic approach, with parameters such as cleavage timing linked to euploidy (Campbell *et al.*, 2013; Zhan *et al.*, 2020). However, this relationship is not absolute either (Rienzi *et al.*, 2015; Barnes *et al.*, 2023). For instance, optimal morphokinetics do not guarantee euploidy, and vice-versa. In this study, euploidy rates were 61.1% for embryos graded as “A” (KIDScore > 7), and 28.0% for “C” graded embryos (KIDScore<4), demonstrating the limitations of morphokinetic scores alone.

This study introduces ICM/TE fragmentation as a novel parameter. Embryos were classified as NN (no fragmentation), FN (ICM fragmentation), or FF (ICM and TE fragmentation). Euploidy rates were 62.5%, 20.1%, and 6.4% for NN, FN, and FF embryos, respectively, demonstrating the value of this parameter. This study achieved area under the curve (AUC) values for euploidy prediction, adjusted for maternal age, of 0.697, 0.688, and 0.791 for KIDScore, Gardner criteria, and ICM/TE fragmentation, respectively. These values indicate that ICM/TE fragmentation demonstrated better predictive capabilities than existing systems. For comparison, Kato *et al.* (2023) reported AUCs of 0.666, 0.655 and 0.642 for IDAScore, KIDScore and Gardner criteria (Kato *et al.*, 2023). A recent meta-analysis using artificial intelligence for euploidy prediction yielded a combined AUC of 0.80 (95% CI 0.76 to 0.83) (Xin *et al.*, 2024), consistent with our findings. Furthermore, odds ratios (ORs) for lower-grade classifications were 0.278, 0.335, and 0.047 for KIDScore, Gardner criteria, and ICM/TE fragmentation, indicating reduced euploidy probabilities of 72.2%, 66.5%, and 95.3%, respectively. Conversely, ORs for aneuploidy associated with lower grades were 3.593, 2.986, and 21.036, translating to 2.6-fold, 2.0-fold, and 20.0-fold increased odds of aneuploidy, respectively. These results align with prior studies showing associations between TE grading and euploidy/aneuploidy rates (Minasi *et al.*, 2016; Wang *et al.*, 2018).

Analysis of aneuploidies showed low-grade embryos had significantly higher rates of chaotic aneuploidy, particularly in Gardner grade C, compared with grade A. This was mirrored in ICM/TE fragmentation analysis, with FF embryos exhibiting a markedly increased frequency of chaotic aneuploidy compared with NN embryos. Monosomy predominated over trisomy in FN and FF embryos, whereas NN embryos exhibited a more balanced distribution of monosomies and trisomies, consistent across all Gardner grades. Prior studies demonstrate increased rates of apoptosis in aneuploid TE cells relative to the ICM (Starostik *et al.*, 2020; Martin *et al.*, 2023; Regin *et al.*, 2024; Zhai *et al.*, 2024) and poorer developmental outcomes in monosomic blastomeres (Shahbazi *et al.*, 2020).

Morula compaction was also investigated (Lagalla *et al.*, 2017, 2020). Partially compacted morulae (Exc-PCM) yielded lower morphological grades than fully compacted morulae (FCM), consistent with previous findings (De Martin *et al.*, 2024; Parriego *et al.*, 2024), but euploidy rates were similar. Morphology disparities may be due to excluded blastomeres compressed against the zona pellucida, resulting in a smoother TE appearance. Paradoxically, many FN and FF embryos originated from FCM, suggesting two subtypes within Gardner grade C: FCM-derived embryos with apoptotic events, and Exc-PCM-derived embryos with poorer morphology but potentially better prognosis. The clinical significance of these subtypes is underlined by studies reporting positive outcomes following the transfer of lower-quality embryos, particularly in patients with a poor prognosis (Cimadomo *et al.*, 2019; Hill *et al.*, 2020; Wang *et al.*, 2020; He *et al.*, 2024). These results support the hypothesis that many viable embryos are likely from the Exc-PCM group, emphasizing the importance of incorporating morula compaction as an additional parameter in embryo selection.

Spontaneous blastocyst collapses (Marcos *et al.*, 2015; Sciorio & Meseguer, 2021; Cimadomo *et al.*, 2022), categorized as minor or major, were investigated for predictive value. Minor collapses were more frequent, with limited predictive value for euploidy. However, increased collapse frequency correlated with higher fragmentation levels, especially in FF embryos.

Embryo selection using morphology or morphokinetics faces limitations in identifying meiotic aneuploidies (Fragouli *et al.*, 2014; Reignier *et al.*, 2018; Tartia *et al.*, 2022). While our system has similar limitations, it shows strong correlations between ICM/TE fragmentation and monosomies/complex abnormalities. Euploidy rates remained consistently low in FN and FF embryos, regardless of morphology. Therefore, embryos without fragmentation could be prioritized for transfer or PGT-A.

There are limitations to the present study that warrant further consideration. This retrospective, single-center study used PGT-A results as a ground truth (Bamford *et al.*, 2023; Barnes *et al.*, 2023) and exclusively included embryos with sufficient quality for biopsy. Furthermore, not all embryos underwent PGT-A, particularly in younger women. This selection bias may explain the higher proportion of fully compacted morulae (73%) observed in this study compared with those of 51% (Parriego *et al.*, 2024) and 57% (De Martin *et al.*, 2024), leading to a potential underestimation of ICM/TE fragmentation.

Despite these limitations, this study offers significant strengths, including the standardization of qualitative parameters and the exclusive use of time-lapse imaging, which enables non-invasive embryo assessment. The novel parameters introduced here enhance the precision of embryo selection and hold promise for integration into automated systems, reducing operator variability and supporting broader clinical adoption.

## CONCLUSION

This study demonstrates that a dynamic, event-based approach effectively complements traditional blastocyst assessment. The association between blastocyst collapse and subsequent ICM/TE fragmentation emerges as a valuable marker suggestive of monosomies or complex chromosomal abnormalities. In addition, the absence of ICM/TE fragmentation, along with tracking morula compaction patterns, may help identify embryos with developmental potential that are otherwise morphologically underestimated. The Blastocyst Fragmentation Indicator (BFI) provides embryologists with a practical tool to refine embryo selection and gain deeper insight into blastocyst development. Further prospective validation is needed to determine the clinical utility of BFI, particularly in improving the non-invasive identification of embryos with higher implantation potential.
